# Guideline-directed device therapies in heart failure: A clinical practice-based analysis using electronic health record data

**DOI:** 10.1016/j.ahjo.2022.100139

**Published:** 2022-05-04

**Authors:** Anne B. Curtis, Christopher Manrodt, Luke D. Jacobsen, Dana Soderlund, Gregg C. Fonarow

**Affiliations:** a Jacobs School of Medicine and Biomedical Sciences, University at Buffalo, Buffalo, NY, United States of America; b Medtronic, Inc., Mounds View, MS, United States of America; c Ahmanson-UCLA Cardiomyopathy Center, Ronald Reagan UCLA Medical Center, Los Angeles, CA, United States of America

**Keywords:** Guideline-directed device therapy, Implantable cardioverter defibrillator, Cardiac resynchronization therapy, Electronic health record, Heart failure

## Abstract

**Background:**

Guideline-directed device therapies (GDDT) improve outcomes for eligible patients with heart failure (HF) with reduced ejection fraction (HFrEF). Utilization rates of device therapies in HFrEF after the 2012 ACCF/AHA/HRS Focused Update for Device-based Therapies of Cardiac Rhythm Abnormalities have not been well studied.

**Objective:**

Characterize the use of GDDT in newly indicated HFrEF patients from 2012 to 2019 using aggregated electronic health record (EHR) data.

**Methods:**

Computable phenotyping algorithms for implantable cardioverter defibrillator/cardiac resynchronization therapy-defibrillator (ICD/CRT-D) indications from the GuideLine Indications Detected in EHR for Heart Failure program (GLIDE-HF) used diagnoses, procedures, measures, prescriptions, and the output of natural language processed provider notes from de-identified Optum® EHR data. Patients had a diagnosis of HF, dilated cardiomyopathy, or prior infarct, and were included if they had HFrEF with >1 year of records prior to a new Class 1 or Class 2a indication for an ICD or cardiac resynchronization therapy with defibrillator (CRT-D) from 2012 to 2019.

**Results:**

Records showed 137,476 HFrEF patients were newly indicated for an ICD or CRT-D. GDDT was used in 14,892 of 36,358 (41.0%) CRT-D indicated patients and in 14,904 of 101,118 (14.7%) ICD-indicated patients. While GDDT use was low, 95.7% had echocardiography and 92.1% had prescriptions for beta-blockers or angiotensin-converting enzyme/angiotensin-receptor blockers medications.

**Conclusions:**

In this modern cohort of HF patients, a large proportion of eligible patients did not receive ICDs or CRT-Ds, while frequently receiving other indicated cardiovascular interventions and treatments.

## 1 Introduction

An estimated 3.2 million United States patients have a diagnosis of heart failure (HF) with reduced ejection fraction (HFrEF) [1]; these patients have an approximate annual mortality rate of 11–14% [2], [3], [4]. Outcomes from many randomized trials inform the guidelines [5] for devices in patients with HFrEF. Quality of care, defined as clinical adherence to treatment guidelines, remains an important area of study in HF [6], [7]. Guideline-directed device therapy (GDDT) in eligible patients has been utilized at lower rates than other guideline-directed interventions in HF [8], [9], [10]. Previous studies of GDDT utilization in HFrEF have been conducted with large-scale manual chart reviews [10] or analysis of insurance claims in treated patients [11], [12], [13]. Trends in device implantation have also been reported from claims data [14]. However, to date there have been only a few studies assessing GDDT in the United States following the ACC/AHA/HRS Focused Update for Device-based Therapy in 2012 or more recent guideline updates.

Aggregated electronic health record (EHR) data collected during routine care show promise as sources of evidence to measure quality of care and to potentially improve patient care [15], [16]. New methods to analyze EHR may be able to address questions where a prospective study is not practical or feasible as well as integrate process-of-care measurements without secondary chart abstraction [17]. However, there is no consensus yet on the best methods to translate EHR data for clinical research and quality assessment [18], [19], [20]. Generating computable phenotypes from EHR data may facilitate quality assessment and improvement efforts.

The objective of this analysis was to assess the utilization of GDDT in HFrEF among patients with a new GDDT indication from 2012 to 2019, using data from a large database of EHR records in the United States.

## 2 Methods

This study was conducted as part of the Guideline Indications Detected in EHR for Heart Failure program (GLIDE-HF). To identify patients eligible for GDDT in HFrEF from the EHR, computable phenotypes [21] were applied to a database of patient records curated by Optum®. Computable phenotypes are algorithms based on the EHR to determine a patient's clinical characteristics. This approach allowed us to generate meaningful results using the Optum EHR database with millions of patients without needing to individually examine provider notes, billing codes, and patient history records. The development and cross-validation of computable phenotypes for ICD indications have been reported separately [22].

The Optum EHR database contains over 30 million patients with diagnosis or procedure records related to cardiovascular disease from more than 60 provider networks in the United States. In addition to ICD-9/10 diagnosis and procedure codes, this database contains common elements included in EHR data, visit records, patient demographics, lab records, prescription records, drug administration records, and information extracted from physician's notes via natural language processing.

Patients were screened first for a diagnosis of HF, dilated cardiomyopathy, or evidence of a prior acute myocardial infarction. The study cohort included only those screened patients with a left ventricular ejection fraction (LVEF) ≤35% who had records meeting a Class 1 or 2a indication for an implantable cardioverter-defibrillator (ICD) or cardiac resynchronization therapy with defibrillator (CRT-D) from 2012 to 2019. Indicated patients were also required to have at least one year of records prior to the first indication, have no prior ICD/CRT-D device, and be 78 years of age or younger. Patients were excluded if they had a prior diagnosis of dementia, brain death, or kidney disease treated by hemodialysis. Having one year of records prior to the first indication helps ensure detection of patients with a prior ICD/CRT-D device or any exclusion diagnoses. Patients who died within 30 days of indication were also excluded.

Class 1 and Class 2a guideline recommendations for ICDs were condensed into three groups: (i) secondary prevention of sudden cardiac death, (ii) primary prevention of sudden death, and (iii) prevention of sudden death after suspected life-threatening ventricular arrhythmias in patients with known arrhythmogenic cardiomyopathies and risk factors. CRT-D recommendations were similarly condensed into four groups: (i) Class 1 recommendation with QRS duration >150 ms and a diagnosis of left bundle branch block (LBBB), (ii) Class 2a recommendation with QRS duration 120-149 ms and a diagnosis of LBBB, (iii) Class 2a recommendation with QRS duration ≥150 ms and without a diagnosis of LBBB, and (iv) other Class 2a recommendations for CRT-D, including those indicated for an ICD with suspected need for >40% bradycardia pacing of the right ventricle. Patients with an LVEF ≤35% at baseline who then have documentation of an LVEF ≥40% are not considered eligible for an ICD unless a subsequent measurement of LVEF is again ≤35%. Patients with a revascularization procedure before indication date are not classified as indicated until 90 days after the procedure date. Similarly, patients with an MI before indication date are not classified as indicated until 40 days after date of MI. As a result, if a patient does not have any records 90 or 40 days after a revascularization or MI respectively, they are not considered eligible for ICD or CRT-D.

Patient groups were assigned distinctly and hierarchically. Patients meeting a CRT-D recommendation prior to any device procedures were assigned to a CRT-D group. The remaining patients were assigned to ICD groups in the order described above. Group assignments and hierarchy were reviewed by cardiologists familiar with the guidelines. Supplemental Fig. 1 summarizes how the cohort was identified and the process for determining indications. Results are reported as simple proportions of device treated and untreated patients.

EHR data reflect clinical standard care not directly related to research, so baseline characteristics desired by researchers post-hoc are not always available in all patients. Additionally, some clinical information in notes may not be captured in the EHR database due to the limitations of natural language processing algorithms and the variability in clinician notes. While it was not feasible to conduct individual chart reviews in the Optum database, other analyses to understand the degree of under-capture of clinical information were conducted. These analyses included reviewing the use of guideline-directed medical therapy in HFrEF patients and comparing baseline characteristics with studies of similar patients.

### 2.1 Endpoints

Patients were marked as treated from the date of the first procedure code for an implant or interrogation of an ICD or CRT-D or record of an ICD or CRT-D implant in Medtronic Device and Registrant Tracking. A patient with a CRT-D indication was considered treated upon any code for an ICD or CRT-D, not just those codes specific to CRT-D. For comparison with other guideline recommendations common to these patients, additional endpoints included prescriptions for beta-blockers, angiotensin-converting enzyme/angiotensin-receptor blockers, as well as an echocardiogram, diagnostic catheterization, and percutaneous coronary interventions or coronary artery bypass graft surgery.

### 2.2 Statistical analysis

Univariate statistics of baseline characteristics were assessed on all patients included in the analysis, done separately for ICD and CRT-D indications, and compared with published baseline data from registries of similar patients. For quantitative variables, means and standard deviations were calculated. The percentage of patients that received ICD or CRT-D implantation was calculated by indication. Also, the percentage of patients with additional HF-related procedures or interventions were calculated.

### 2.3 Ethical approval

The Optum database contains fully deidentified data and was not classified as research involving human participants. Accordingly, institutional review board approval was not required.

## 3 Results

**Fig. 1:**
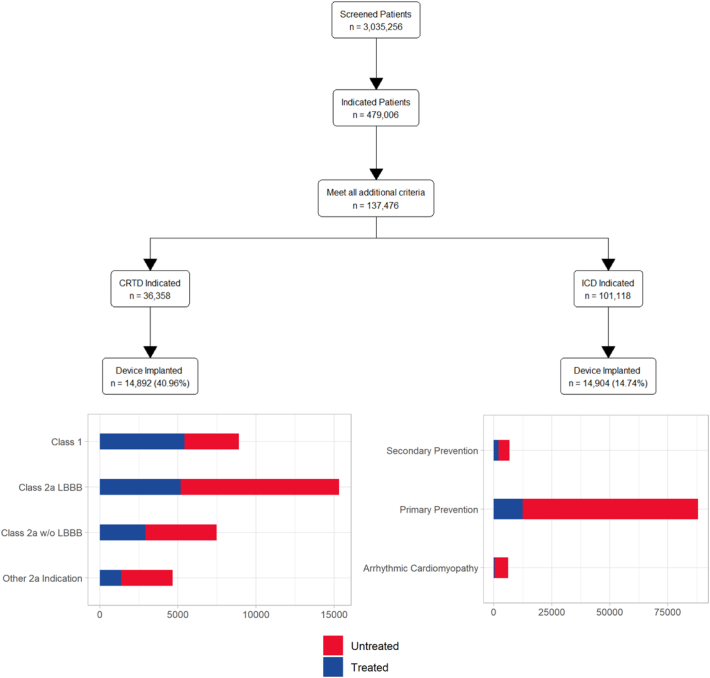
Use of Guideline-directed Devices in HFrEF. The top flow chart shows the patients selected from each stage and the final cohort included in the analysis. The bottom right bar graph shows the distribution of the ICD eligible patients stratified by indication. The bottom left bar graph shows the distribution of the CRT-D eligible patients stratified by indication. *Note: Treated patients had received any defibrillator device, including ICD, CRT-D or S-ICD HF = Heart Failure, CRT-D = Cardiac Resynchronization Therapy Defibrillator, HFrEF = Heart Failure with reduced Ejection Fraction, ICD = Implantable Cardioverter Defibrillator, LBBB = Left Bundle Branch Block S-ICD = Subcutaneous Implantable Cardioverter Defibrillator.

There were 3.04 M patients who met screening criteria. After screening, 479,006 patients had evidence of HFrEF and an indication for an ICD or CRT-D. From these patients, a total of 137,476 patients were included in the analysis, after limiting patient inclusion to the first date of indication in 2012–2019 with at least one year of prior records free of device-related procedures, and after excluding patients for early mortality, old age, and contraindicated diagnoses (Fig. 1, top flow chart).

Records of a CRT-D indication were present in 36,358 patients, and 14,892 (41.0%) received GDDT (Fig. 1, middle left). Among the remaining 101,118 patients who met an ICD indication, 14,904 (14.7%) were implanted with GDDT (Fig. 1, middle right). Device use varied among CRT-D indicated patients, from 5423 of 8900 (60.9%) with Class 1 indications and 2928 of 7481 (39.1%) with Class 2a indications without LBBB, to 5175 of 15,313 (33.8%) with Class 2a with LBBB (Fig. 1, bottom left).

Most ICD-eligible patients were in the primary prevention group, with 12,467 of 88,103 (14.2%) patients receiving devices. Among patients in the secondary prevention group, 2037 of 6750 (30.2%) were implanted with GDDT (Fig. 1, bottom right).

### 3.1 Diagnostic testing and medical therapy

**Fig. 2:**
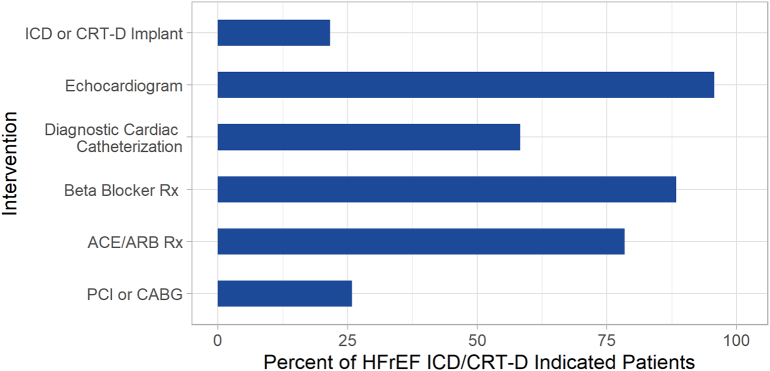
Other treatments in GDDT-indicated patients. The percent of HFrEF indicated for either an ICD or CRT-D are shown by the interventions for HF that they received. *ACE/ARB = angiotensin-converting enzyme inhibitor system antagonists/Angiotensin II Receptor Blockers, CABG = Coronary Artery Bypass Graft, CRT-D = Cardiac Resynchronization Therapy Defibrillator, GDDT = Guideline Directed Device Therapy HF = Heart Failure, HFrEF = Heart Failure with reduced Ejection Fraction, ICD = Implantable Cardioverter Defibrillator, PCI = Percutaneous Coronary Intervention, Rx = Medical Prescription.

Among all patients in this study, 95.7% had echocardiography and 92.1% had prescriptions for beta blockers or angiotensin-converting enzyme/angiotensin-receptor blockers medications. While not all patients in this study were expected to be indicated for the following procedures, 58.3% had diagnostic catheterizations, and 25.8% had percutaneous coronary interventions or coronary artery bypass graft procedures (Fig. 2).

## 4 Discussion

This large, multi-year analysis of GDDT in patients with HFrEF in clinical practices showed much lower utilization than what would be expected based on published guidelines, and lower utilization than earlier published reports [10]. While device use was similar to a recent large drug trial in HFrEF [9], these low utilization rates are striking, despite “the voluminous literature related to the efficacy of these devices in the treatment and prophylaxis of sudden cardiac death and malignant ventricular arrhythmias” [5] cited in the guidelines. Prior studies have shown underutilization of ICD and CRT-D devices for primary prevention of sudden cardiac death in eligible patients with HFrEF [10]. The Registry to Improve the Use of Evidence-Based Heart Failure Therapies in the Outpatient Setting (IMPROVE-HF) study was an outpatient quality improvement study that included data on baseline utilization of ICDs and CRT-D in heart failure patients and improvement in utilization at 24 months after implementation of a quality improvement program. Baseline utilization was 50.1% for ICD/CRT-D and 77.5% at 2 years, and utilization of CRT-P/CRT-D was 37.2% at baseline and 66.3% at 2 years. While these were significant improvements in utilization, there was still a gap in utilization that suggests further efforts are needed for patients to receive indicated device therapy [10]. Like IMPROVE-HF, this study also found wide variations in GDDT use across practices.

One advantage of the current analysis is that the EHR enabled obtaining data for all GDDT-eligible patients across all practice settings in these large healthcare systems. Unlike registry studies that depend on referrals to investigators, this analysis may offer insight into a wider group of device-eligible HFrEF patients.

### 4.1 Primary prevention ICD patients have the lowest utilization despite a Class 1 recommendation

Unsurprisingly, CRT-D use was higher than ICD use. Device use varied substantially among indication groups. CRT-D Class 1 had the highest use, followed by CRT-D Class 2a subgroups and secondary prevention ICDs. The lowest use occurred in the largest group, primary prevention ICDs at 14.2%, despite a Class 1 recommendation.

### 4.2 Baseline characteristics similar to other HFrEF studies

To assess external consistency, patient characteristics were compared with two multi-center registry studies in HFrEF (Table 1). ACTION HF enrolled patients with ambulatory HF, New York Heart Association Class 3–4, and LVEF <35% [23]. Age, ischemic etiology, and ejection fraction were similar. The European Society of Cardiology Heart Failure Long-Term Registry, a prospective observational study, is collecting epidemiological information and 1-year follow-up data in 9134 HF patients [24]. The subgroup of patients with LVEF <40% has comparable baseline characteristics as well.

**Table 1:** Patient characteristics.

	ICD indicated (n = 101,118)	CRT-D indicated (n = 36,358)	HF-ACTION (n = 2331)	ESC HF LT (HFrEF) (n = 5460)
Age, years	61.3 ± 11.7	65.2 ± 10.4	59*	64 ± 12.6
Male	64.9%	64.4%	71.6%	78.4%
Race				
Asian	0.8%	0.6%		
Black	18.6%	13.2%		
Hispanic	3.7%	3.9%		
Other	2.9%	2.4%		
White	74.0%	79.9%		
Diabetes	37.5%	43.1%	32.0%	32.3%
Ischemic	44.9%	51.0%	51.3%	48.6%
LVEF	25.3 ± 7.9	24.9 ± 7.7	25*	29.1 ± 7.6

CRT-D = Cardiac Resynchronization Therapy Defibrillator, ESC HF LT (HFrEF) = European Society of Cardiology Heart Failure Long-Term Registry (Heart Failure with reduced Ejection Fraction), HF-ACTION = Heart Failure and A controlled Trial Investigating outcomes of Exercise TraiNing, ICD = Implantable Cardioverter Defibrillator, LVEF = Left Ventricular Ejection Fraction.

### 4.3 Impact of underestimation of eligible patient population

Our analysis was restricted to patients who had specific criteria in their EHR. Patients in our analysis had records showing a low ejection fraction, and the vast majority had received prescriptions for guideline-directed medical therapy. These patients met the other criteria that accompany a low LVEF for device eligibility. Patients also showed comorbidities typical of device-eligible HFrEF patients. Guideline recommendations were implemented directly into computable phenotypes from the EHR, without imputation. The risk of missing data would potentially underestimate the population of eligible patients. And yet, even among those patients where all criteria were directly present in the records, utilization of GDDT was markedly lower than utilization of other guideline-recommended therapy.

### 4.4 Future studies identifying methods to increase adoption necessary

Despite clinical trial evidence and national guidelines recommending ICD and CRT-D placement for eligible patients with HFrEF, these contemporary data suggest there are substantial opportunities to improve use among eligible patients. One potential reason for the difference in utilization among therapy types is the degree of therapy invasiveness. More invasive procedures often introduce additional issues of patient willingness, feasibility, cost, and others that need to be addressed to increase utilization of GDDT. More education and emphasis on performance improvement systems to facilitate adoption of guidelines in HFrEF are needed. The IMPROVE-HF performance improvement program was associated with an increase in ICD use of 30.3% and CRT-D use of 30.8% over the course of 24 months [10]. National guidelines recommend the use of programs to identify appropriate patients for therapy, to provide practitioners with useful reminders based on the guidelines, and to continuously assess the success achieved in providing these therapies to patients who can benefit from them [25]. The demonstration that there remain substantial gaps in the use of ICD and CRT-D therapy suggests there is an important need to better implement these recommendations. These findings also suggest that an ICD and CRT-D process-of-care performance measure might be helpful in reducing gaps, variations, and disparities in the use of ICDs and CRT-D in clinical practice.

### 4.5 Limitations

This study has limitations that could influence the estimation of device use. First, the data source is a non-randomized sample of 30 million patients with diagnosis or procedure records related to cardiovascular disease from this over 60 provider network. Therefore, since this is a non-randomized sample, the data may not represent the real-world trends of the HFrEF population in the United States. Because of the nature of the Optum database, this analysis was not able to be validated with medical source documentation review. However, future analyses should consider, when possible, detailed chart review for identifications of indications and exclusions. The Optum database overrepresents patients from the Midwest and underrepresents patients from the South, which could potentially skew demographics. Also, many key criteria for indications could only be derived from natural language processed data, such as QRS durations and HF symptoms of NYHA Class 2 or 3. Patients were only included if these data were detected. In the previously reported analysis [22], among patients who received devices and had at least one year history of EHR, 11.9% had insufficient records to identify an ICD indication. These missing data suggest the potential for an over-estimation of device use.

Conversely, there is the potential for a patient to have EHR data that provide evidence of an indication and receive a device outside of the Optum network, resulting in an under-estimate of device use. Considering procedure codes for device interrogation and including device implant data from Medtronic Device and Registrant Tracking as evidence of device implantation help mitigate this limitation. Additionally, while computable phenotypes were developed for common cardiovascular and cerebrovascular comorbidities, algorithms were not developed for all possible conditions that would lead to “life expectancy less than one year” or lead the risk:benefit ratio of ICD/CRT-D implantation to become unfavorable, such as metastatic cancer. Further algorithm development may have identified and excluded some additional patients. The broad exclusion based on age attenuates this effect.

## 5 Conclusions

In this contemporary cohort of HF patients, a substantial proportion of indicated patients did not receive ICDs or CRT-D. Further, the ICD and CRT-D treatment rates are lower compared to other cardiovascular interventions and treatments, including drug therapies. This evidence suggests there is a substantial opportunity to increase GDDT in eligible patients. Finally, our results suggest that further research into the factors associated with device use in patients with guideline indications is warranted.

## 6 Clinical perspectives

### 6.1 Competency in patient care

In this retrospective, observational analysis of 137,476 patients with heart failure and reduced left ventricular ejection fraction (HFrEF), guideline-directed device therapy (GDDT) was low in eligible patients. Device use was lowest among patients meeting a Class 1 indication for primary prevention of sudden death. Nearly all patients had records of cardiac imaging and guideline medical therapy, suggesting that barriers to devices in HFrEF may be different than other guideline interventions.

### 6.2 Translational outlook 1

There is a substantial opportunity to increase device use in eligible guideline-indicated patients, especially for prevention of sudden cardiac death in HFrEF populations.

### 6.3 Translational outlook 2

Further research into the factors associated with device use and methods to increase device therapy adoption in indicated patients is warranted.

The following is the supplementary data related to this article.

Supplemental Fig. 1 Summary of cohort identification and the process for determining indications. Supplemental Fig. 1

## Funding

This work was supported by 10.13039/100004374Medtronic Inc. as the sponsor of the GLIDE-HF program.

## Declaration of competing interest

The authors declare the following financial interests/personal relationships which may be considered as potential competing interests:

CM, LDJ, & DS: Employee/Shareholder – Medtronic, Inc.; ABC: Honoraria/Speaking Fee and data monitoring board for clinical trial – Medtronic, Inc.; advisory board: Janssen Pharmaceuticals, Abbott, Sanofi Aventis, Milestone Pharmaceuticals; honoraria for speaking: Abbott; GCF: Consulting – Abbott, Amgen, CHF Solutions, Janssen, Medtronic, Inc., Merck, and Novartis.
