# Supplementary material for: Guideline-directed device therapies in heart failure: A clinical practice-based analysis using electronic health record data
Source: Am Heart J Plus. 2022 May 4;16:100139. doi: 10.1016/j.ahjo.2022.100139 (PMC10976280; doi:10.1016/j.ahjo.2022.100139)
Supplement: Supplemental Fig. 1 — Summary of cohort identification and the process for determining indications. [file mmc1.pptx]

## Slide 1
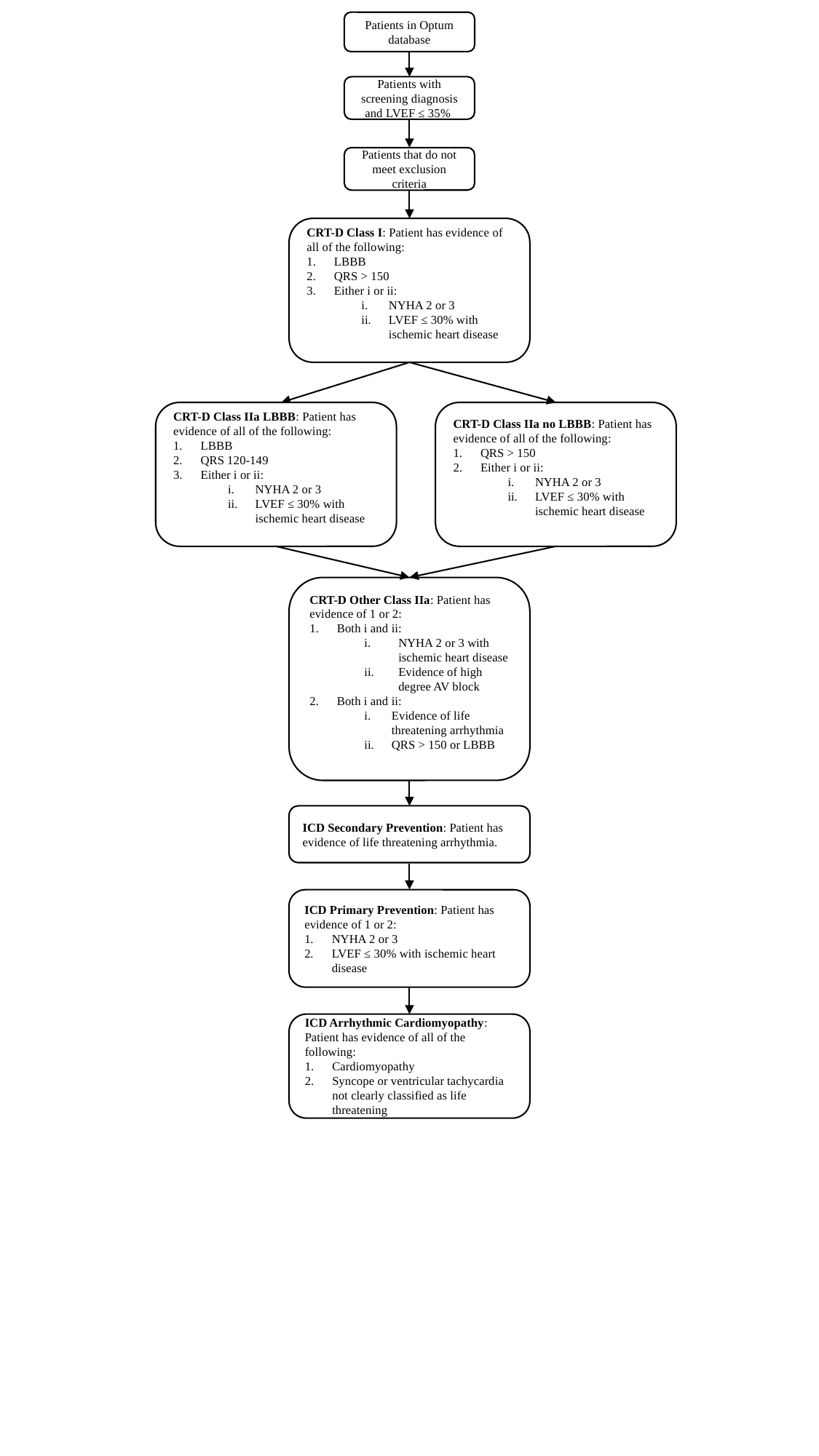

Patients in Optum database
Patients with screening diagnosis and LVEF ≤ 35%
Patients that do not meet exclusion criteria
CRT-D Class I: Patient has evidence of all of the following:
LBBB
QRS > 150
Either i or ii:
NYHA 2 or 3
LVEF ≤ 30% with ischemic heart disease
CRT-D Class IIa LBBB: Patient has evidence of all of the following:
LBBB
QRS 120-149
Either i or ii:
NYHA 2 or 3
LVEF ≤ 30% with ischemic heart disease
CRT-D Class IIa no LBBB: Patient has evidence of all of the following:
QRS > 150
Either i or ii:
NYHA 2 or 3
LVEF ≤ 30% with ischemic heart disease
CRT-D Other Class IIa: Patient has evidence of 1 or 2:
Both i and ii:
NYHA 2 or 3 with ischemic heart disease
Evidence of high degree AV block
Both i and ii:
Evidence of life threatening arrhythmia
QRS > 150 or LBBB
ICD Secondary Prevention: Patient has evidence of life threatening arrhythmia.
ICD Primary Prevention: Patient has evidence of 1 or 2:
NYHA 2 or 3
LVEF ≤ 30% with ischemic heart disease
ICD Arrhythmic Cardiomyopathy: Patient has evidence of all of the following:
Cardiomyopathy
Syncope or ventricular tachycardia not clearly classified as life threatening
